# A novel application of statistical process control charts in financial market surveillance with the idea of profile monitoring

**DOI:** 10.1371/journal.pone.0288627

**Published:** 2023-07-20

**Authors:** Ali Yeganeh, Sandile Charles Shongwe

**Affiliations:** Faculty of Natural and Agricultural Sciences, Department of Mathematical Statistics and Actuarial Science, University of the Free State, Bloemfontein, South Africa; URV: Universitat Rovira i Virgili, SPAIN

## Abstract

The implementation of statistical techniques in on-line surveillance of financial markets has been frequently studied more recently. As a novel approach, statistical control charts which are famous tools for monitoring industrial processes, have been applied in various financial applications in the last three decades. The aim of this study is to propose a novel application of control charts called profile monitoring in the surveillance of the cryptocurrency markets. In this way, a new control chart is proposed to monitor the price variation of a pair of two most famous cryptocurrencies i.e., Bitcoin (BTC) and Ethereum (ETH). Parameter estimation, tuning and sensitivity analysis are conducted assuming that the random explanatory variable follows a symmetric normal distribution. The triggered signals from the proposed method are interpreted to convert the BTC and ETH at proper times to increase their total value. Hence, the proposed method could be considered a financial indicator so that its signal can lead to a tangible increase of the pair of assets. The performance of the proposed method is investigated through different parameter adjustments and compared with some common technical indicators under a real data set. The results show the acceptable and superior performance of the proposed method.

## 1. Introduction

Statistical process control (SPC) is a well-known technique in industrial process for the reduction of non-compatible products, elimination of adverse conditions and generally improving the final quality of a process [1]. It includes seven main techniques and tools, these are: cause-and-effect diagram, Pareto chart, control chart, histogram, scatter diagram, check sheet and stratification; by which a specific process is monitored and controlled. Control charts were proposed in the third decade of twenty century by Walter A. Shewhart and are the most useful SPC tools for the aim of industrial process monitoring [2]. After that several monitoring schemes have been extended for symmetric and asymmetric distributions by means of control charts in the area of process monitoring. Although industrial process has attracted the most attention, there are some other applications in which control charts have been successfully applied for monitoring vital characteristics over the time. To name just a few, we refer readers to a study based on customer complaints monitoring [3], social network surveillance [4], health-care process [5], highway safety surveillance [6], and several other fields [7,8].

As a different application, control charts have been widely implemented in different areas of the economic and financial market monitoring (this area of study is sometimes referred to as financial surveillance). Bisiotis, Psarakis [9] have provided a comprehensive review of this subject in which some general categories could be found. One major category is about monitoring of the portfolio weights with the aid of control charts. In this approach, control charts aid the investors in a better trade-off between the expected return and the risk of an investment. Golosnoy and Schmid [10] utilized two well-known control charts, i.e., Hoteling T^2^ (hereafter denoted by T^2^) and Exponentially Weighted Moving Average (EWMA). The T^2^ and EWMA charts were used as methods for portfolio weights adjustment in a multivariate monitoring setup. They also modified the control charts by minimization of ex-ante portfolio’s variance. An excellent account of this work in terms of the detection ability was proposed by Golosnoy, Okhrin [11]. The performance of another well-known control chart, i.e., CUmulative-SUM (CUSUM) was also evaluated in this field of study by Golosnoy, Ragulin [12]. As a novel approach to portfolio weights adjustment, Riegel Sant’Anna, Pascoal Filomena [13] combined the control chart idea with a rebalancing strategy and index tracking in which a group of assets is chosen to monitor the market and portfolio. More details about the index tracking technique were provided by Nesi Bubicz, Pascoal Filomena [14].

Modeling of financial surveillance with Auto Regressive Moving Average (ARMA) and Generalized Auto Regressive Conditional Heteroskedasticity (GARCH) time series has become a fundamental approach in such a way that the control charts are utilized to monitor the model’s weights or residuals. Kovářík and Klímek [15] evaluated EWMA and CUSUM control charts in the financial time series and showed that it was highly likely to see the autocorrelation effect in financial time series which caused inappropriate performance of control charts. To remedy this challenge, they suggested monitoring residuals of the ARMA model instead of the raw (original) values of financial key characteristics. Similar ideas could also be found in Kovářík, Sarga [16], Sadeghi, Owlia [17] and Golosnoy and Roestel [18]. In addition to the autocorrelation effect, control charts in financial time series may suffer from two other undermining factors, i.e., non-normality of observations that causes asymmetric distribution of errors, and data generation delay by which the control chart loses its real performance. For these situations, Pérez-Rave, Muñoz-Giraldo [19] indicated that monitoring the residuals could be considered as a reasonable solution in a real problem in the transport logistics sector. As a different approach, Doroudyan and Niaki [20] considered an ARMA-GARCH time series model to implement a pattern recognition problem with the aim of machine learning techniques. In fact, they utilized machine learning techniques as a control chart instead of statistical approach.

Another important category of control charts applications in financial markets is about triggering some signals about the market conditions. In this category, it is of much importance to identify the assignable cause (or shifts) in the stable (normal) condition, which is also known as the In-Control (IC) state in the SPC literature, as soon as possible to avoid failure in investments. Golosnoy [21] monitored the beta parameter (i.e., which is a factor of asset returns) by a univariate Shewhart control chart and a T^2^ scheme. The signals indicated the days which had different portfolio betas from the investors’ demands. Hassan, Kumiega [22] defined a trading system based on the EWMA control chart. To interpret each signal as bearish or bullish, they provided some rules and conditions based on the experts’ opinions. The 30-year observations of US Dollar (USD), Euro, and Japan Yen were monitored with EWMA and CUSUM control charts in Garthoff, Golosnoy [23]. They used a GARCH model with consideration of the covariance matrix as a multivariate problem. This procedure was also conducted with Zagreb stock exchange market data in Croatia by Dumičić and Žmuk [24]. They concluded that control charts might not be able to provide satisfactory signals about purchase and buy in the occurrence of autocorrelation. Szetela [25] employed CUSUM and moving range charts for the most important cryptocurrency market (i.e., Bitcoin (BTC)) to compare the volatility rate of the EURO and BTC.

Some other categories of the application of control charts in financial market monitoring were reported in Bisiotis, Psarakis [9]. The study by Bisiotis, Psarakis [9] and other investigations in the related literature revealed that all of them have been extended based on the monitoring quality characteristic (or direct monitoring). Note that an indirect approach called profile monitoring has been developed from the beginning of the twentieth century [26–28], however, it has not been adopted for financial market monitoring. The general idea of the direct approach is based on the definition of a specific distribution or a non-parametric model to monitor the quality characteristics of such a process mean and variance as a univariate or multivariate quality characteristics. For the indirect approach, the monitoring process is well represented by a relationship between the response variable and one or more regressors (explanatory variables) and the major aim of profile monitoring is to monitor the stability of the predefined IC model (or profile) over time [29].

As a brief literature review, the study of Kang and Albin [26] is the pioneer work to develop EWMA and Hoteling T^2^ schemes in linear profiles. They monitored the regression parameters, as well as model’s residuals. After that, Kim, Mahmoud [30] suggested a transformation of the explanatory variables to reach the mean of transformed values as zero and subsequently they utilised three separate EWMA control charts for monitoring intercept, slope and error variance. Zou, Tsung [31] scaled the profile parameters to reach a multivariate EWMA statistic which was called MEWMA. From that time till now, several theorical and practical contributions have been proposed in this area of study. Development of generalized likelihood ratio [32], Bayes theorem [33], random effect model [34], semiparametric methods [35] and machine learning [36] in profile monitoring are some instances for the first group. On the other hand, we can find several practical applications of profile monitoring in different industries such as wood composites [37], chemical gas sensors [38], shrimp farming [39] and so forth. As there are a vast number of research papers related to this topic, it is not possible to mention them all, but we refer the interested readers to the existing review papers by Woodall and Montgomery [40] and Maleki, Amiri [41].

As mentioned, previous research in monitoring financial markets were carried out in the area of monitoring quality characteristics and there are no control charts under the assumption of profile monitoring. On the other hand, except for the work of Szetela [25], after questing the literature, we did not find any other research about monitoring of cryptocurrencies with control charts. In this area of study, any performance comparisons and signal interpretation were not provided by Szetela [25]. It is expected that the signals from a control chart can play a role, as such a financial technical indicator, is used in forecasting the market’s future evolution and direction [42–44] while there is no novel research for this aim and also, some researchers such as Hassan, Kumiega [22], Garthoff, Golosnoy [23], Dumičić and Žmuk [24] and Golosnoy and Roestel [18] stated that the signals could not be applied directly as a technical indicator and it is necessary to consult with the experts for a proper decision. To bridge these knowledge gaps, this study aims to monitor the relationship between the price of assets in a portfolio as a profile monitoring approach. Without loss of generality, the assets are selected from two major cryptocurrencies, i.e., BTC and Ethereum (ETH). Due to the importance of autocorrelation in financial markets [17,19], a novel T^2^ control chart is developed to consider the effect of autocorrelation in the profiles. Also, the signals are interpreted with some rules in such a way that the proposed method could be considered a technical financial indicator and trading machine. Hence, the proposed method of this paper called hereafter Control Chart based Profile Monitoring (CCPM) provides a trading strategy based on the SPC charts for the generation of buy and sell signals which are equivalent to changing the assets from BTC to ETH (for buy) and ETH to BTC (for sell).

The organization of this study is as follows: Section 2 provides the related preliminaries of this study. In Section 3, we discuss the proposed monitoring approach and parameter tuning. Performance comparisons are illustrated in Section 4. Finally, Section 5 summarizes the conclusion and directions for future research.

## 2. Preliminaries

The general concept of SPC charts in the area of profile monitoring is described in the following subsection and then, the problem definition of cryptocurrencies monitoring is illustrated in subsection 2.2. Finally, the common financial technical indicators in the cryptocurrency market are introduced subsection 2.3.

### 2.1. Control charts in profile monitoring

To implement a SPC chart, Phase I and II are defined with different goals. The estimation of parameters and outlier detection from historical dataset are conducted in Phase I; hence, it is sometimes denoted as off-line monitoring. On the other hand, the on-line surveillance of the process for a quick detection of assignable causes is referred to as Phase II (or on-line monitoring). The occurrence of assignable causes, which is also known as Out-Of-Control (OC) condition, leads to the elimination of the predefined Phase I stability conditions in a process so it is expected that a control chart triggers an OC signal as soon as possible in Phase II [45]. In Phase I, the IC parameters (model) are derived from the historical data and then the control charts’ limits are designed. In Phase II analysis, the control chart statistic is computed and compared with the control limits in such a way that the process is terminated in case of an OC signal [40].

There exist several types of profiles, including linear, polynomial, Poisson, logistic, and so forth, among which the simple linear profile model has been frequently extended in the related literature [41]. Suppose that the IC linear model which includes a pair of response (dependent) and explanatory (independent) variables is obtained from a historical data in Phase I with the following parameters:

$$\begin{array}{l} Y_{ij}=A_{0}+A_{l}X_{ij}+\varepsilon_{ij},\\ i=1,2,\ldots ,n,\\ j=1,2,\ldots . \end{array}$$ (1)

At the time of the *j*^*th*^ inspection from the beginning of Phase II, it is assumed that each profile includes *n* pairs (*Y*_*ij*_, *X*_*ij*_) of observations where *Y*_*ij*_ is the response and *X*_*ij*_ is the explanatory variable. Also, the intercept and slope parameters in IC condition are indicated by *A*_*0*_ and *A*_*1*_, respectively, and *ε*_*ij*_ is the independent error term that follows a standard normal distribution with mean 0 and variance *σ*^*2*^. The random and fixed models are possible for the explanatory variable *X*_*ij*_ in Eq (1) depending on the nature of the problem, see the discussion on fixed and random explanatory variables in Noorossana, Fatemi [46], Abbas, Rafique [34], Abbas, Mahmood [38] and Malela-Majika, Shongwe [47]. Noorossana, Fatemi [46] extended some control charts, such as Hotelling’s T^2^, *F* test, EWMA and so forth, for this situation and evaluated their performance. The three papers (i.e., Abbas, Rafique [34]; Abbas, Mahmood [38]; Malela-Majika, Shongwe [47]) proposed some complicated memory type-based techniques to increase the detection ability under the random explanatory variables, some of which are different ranked set sampling, Bayesian prior and posterior definition, Double EWMA (DEWMA), time-varying and asymptotic control limits and so forth. In addition, the index *j* of *X*_*ij*_ indicates that it is allowed to change from one profile to another. At the *j*^*th*^ inspection of Phase II monitoring procedure, the parameter of the statistical model in Eq (1) could be estimated by the Least Square Estimates (LSE) technique as [46]:

$$\begin{array}{l} {\hat{A}}_{1j}=\frac{\sum_{i=1}^{n}Y_{ij}(X_{ij}-{\bar{X}}_{j})}{S_{xx_{j}}},\\ {\hat{A}}_{0j}={\bar{Y}}_{j}-{\hat{A}}_{1j}{\bar{X}}_{j},\\ {\hat{\sigma }}_{j}=\frac{\sum_{i=1}^{n}{\left(Y_{ij}-{\hat{A}}_{0j}-{\hat{A}}_{1j}X_{ij}\right)}^{2}}{n-1}, \end{array}$$ (2)

where ${\bar{X}}_{j}=\frac{\sum_{i=1}^{n}X_{ij}}{n},{\bar{Y}}_{j}=\frac{\sum_{i=1}^{n}Y_{ij}}{n}$ and ${S_{XX}}_{j}=\frac{\sum_{i=1}^{n}{\left(X_{ij}-{\bar{X}}_{j}\right)}^{2}}{n}$. In Eq (2), ${\hat{A}}_{0j}$ and ${\hat{A}}_{1j}$ are the estimators of the intercept (*A*_*0*_) and the slope (*A*_*1*_), respectively.

### 2.2. Problem definition about monitoring cryptocurrency market

After the introduction of BTC in 2009, a novel area of virtual or digital currency appeared in trading systems and the global economy. Although there has been only one cryptocurrency i.e., BTC until 2011, nowadays, there are more than 5000 cryptocurrencies, $90 billion trading volume in a 24-hour interval and 6 million active users in this industry [48,49]. BTC and ETH were able to reach the most famous and interesting assets among a plethora of cryptocurrencies in such a way that they had the largest trading volumes and highest market capitalizations [44]. However, there are significant volatility, unpredictable factors, and excessive price fluctuations in these markets over time in such a way that prediction of their future direction is a very challenging problem [50].

**Fig 1:**
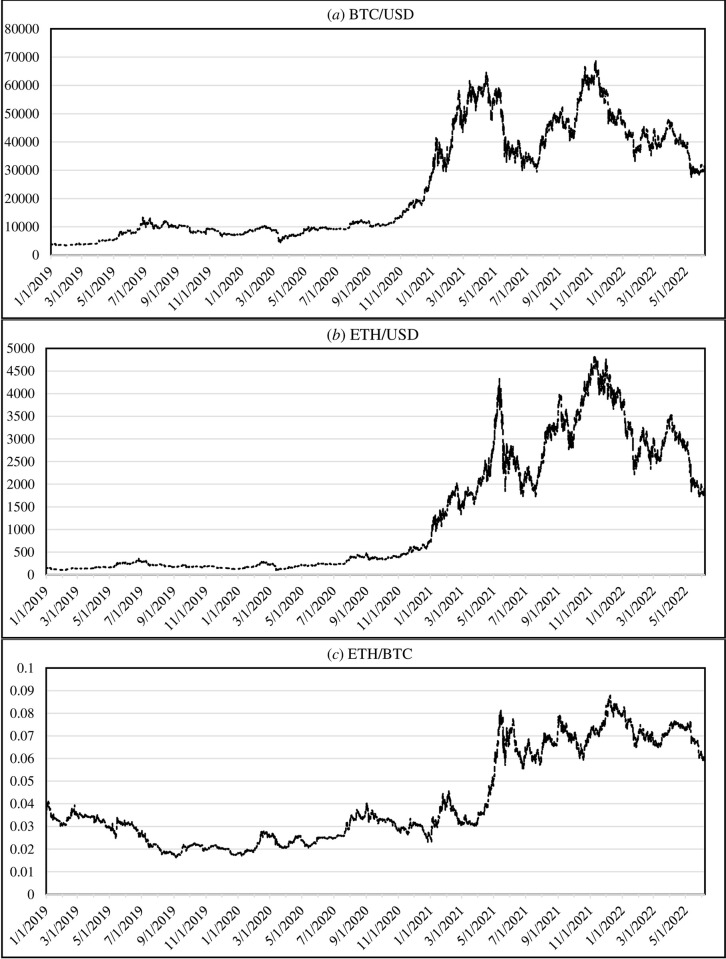
The close price of (a) BTC, (b) ETH and (c) ETH/BTC during the period 1/1/2019 to 07/06/2022 (source: https://www.cryptodatadownload.com).

Several kinds of research have been conducted with the aim of predicting the future direction in this area based on the technical indicators [44,51], social media analysis [52], machine learning [53] and so forth. Most of the previous researches considered a specific cryptocurrency individually, while as a supplementary technique, especially for professional traders, it could be useful to investigate a trading pair, like ETH/BTC. In this approach, traders convert their BTC and ETH to each other instead of using Dollar, Tether, Euro, or some common markets to make benefit from the fluctuations of these two cryptocurrencies. To provide an overall insight, Fig 1 depicts the close price of BTC, ETH and ETH/BTC during the period 01/01/2019 to 07/06/2022. It is noteworthy to mention that the close price was extracted from the popular candlestick chart analysis approach in which four prices, including Open, High, Low and Close (OHLC), provide a graphical plot for the price trend [54].

For better illustration, Table 1 provides a comparison between the obtained benefits in the case of conventional cost variation and implementation of coin conversion. Note that two timestamps, i.e., 15 July 2021 and 9 August 2021, were investigated in Table 1. The BTC and ETH prices were 31835 and 1920 dollars at the first time and 46998 and 3424 dollars at the second time, respectively. It is assumed that there were 0.05 BTC and 0.8 ETH in the wallet, which was nearly about 3128 dollars. Without any action and by holding these values, the total capital had been changed to 5089 dollars (i.e., 63% benefit). However, one can increase this benefit with the conversion strategy. In this approach, 50% of BTC had been changed to ETH in such a way that we had 0.025 BTC and 1.21 ETH (note that on 15 July 2021, 0.025 BTC was equal to 0.41 ETH), the total capital would have reached 5333 dollars (i.e., 71% benefit). Consequently, it could be said that there were 0.05 BTC and 0.87 ETH on 9 August 2021 by applying the proposed strategy. In other words, as the price of ETH/BTC was increased during this time, the conversion of BTC to ETH was a beneficial strategy that was equal to a buy signal. It is obvious that the reverse approach had to apply in the case of the sell signal with ETH/BTC price. It is noteworthy to mention that the variations of the ratio of two cryptocurrencies is important for us in this approach to increase the final value of BTC and ETH as we were able to increase the ETH from 0.8 to 0.87 in this example. In other words, as a possible scenario, the price of both BTC and ETH might be reduced between 15 July 2021 and 9 August 2021 so the total asset was also decreased in terms of USD despite our total BTC and ETH being increased.

**Table 1:** Comparing the obtained benefits between the conventional cost variation and implementation of coin conversion.

Without any signal action						
Date	15-Jul-21			9-Aug-21		
Signal phase	First time			Second time		
Cryptocurrency	BTC	ETH	ETH/BTC	BTC	ETH	ETH/BTC
Unit Price	31835	1920	0.060311	46998	3424	0.072854
Volume	0.05	0.8		0.05	0.8	
Each asset (USD)	1592	1536		2349.9	2739.2	
Total asset (USD)	3128			5089		
With signal action (conversion of coins)						
Cryptocurrency	BTC	ETH	ETH/BTC	BTC	ETH	ETH/BTC
Unit Price	31835	1920	0.060311	46998	3424	0.072854
Volume	0.025	1.21		0.025	1.21	
Each asset (USD)	796	2332		1175	4159	
Total asset (USD)	3128			5333		

### 2.3. Conventional technical indicators in cryptocurrency market

As CCPM is implemented as a technical indicator to predict the future direction of ETH/BTC, it is necessary to compare it with some other conventional financial technical indicators. There are several technical indicators which usually are categorized in five groups as: overlap study, cycle, momentum, volatility and pattern recognition [44]. Following Gruszka and Szwabiński [55], we selected two famous technical indicators, including Relative Strength Index (RSI) and Moving Average Convergence Divergence (MACD). In this subsection, a brief overview of them is presented.

The RSI, which is from the momentum-based oscillator indicators, measures the speed and change (magnitude) of the price direction. For computation of RSI, another common indicator called Exponential Moving Average (EMA) is applied by consideration of a given *N*-days smoothing factor. To this end, an upward and downward change during the *N*-days is computed and then their ratio makes the RSI value. For brevity, the formulas are not given here but the readers can refer to Rodríguez-González, García-Crespo [56]. The possible range of RSI is 0–100 and several interoperations could be found for it. As a common approach, a large (small) value of RSI is the indicator of an overbought (oversold) condition and we also selected this idea for comparison [57].

The MACD is another common momentum-based oscillator indicator that can be calculated subtracting the longer moving average from the shorter one. It includes two major lines as signal line crossovers and center (or MACD) line crossovers to generate signals. The MACD line is usually calculated by subtracting the 26-period EMA from the 12-period moving average. On the other hand, the nine-period EMA of the MACD line is used in the creation of the signal line. By plotting two lines, one can interpret the market direction as a bearish (bullish) condition when the MACD turns down and crosses below (above) the signal line, respectively [58].

## 3. The proposed method

The CCPM approach consists of three steps, including: (*i*) estimation, (*ii*) monitoring, and (*iii*) decision making. To implement these steps, it is required to define a novel chart statistic in this problem. Hence, we first describe our proposed model in subsection 3.1 and then the next subsection illustrates proposed chart statistic and finally each step comprehensively is defined.

### 3.1. The proposed profile model

As the main contribution of this study, we consider the relationship between the coins as a profile and the major aim is to monitor relationship with the SPC profile monitoring techniques. In other words, a target relation between two coins is first obtained from historical data (Phase I) and then, a signal is triggered when the relation does not remain in the IC condition. So, the OC signal is equal to changing the IC formula to an OC relation. After some investigations, it was observed that the simple linear model could reasonably be fitted for these two coins in a short period of time. So, it was decided to monitor the daily relation between the two coins in such a way that the hourly close prices in each day establish the profile of the response and explanatory variables. By this aim, we rewrite Eq (1) as follows:

$$\begin{array}{l} ETH_{ij}=A_{0}+A_{l}BTC_{ij}+\varepsilon_{ij},\\ i=1,2,\ldots ,24,\\ j=1,2,\ldots , \end{array}$$ (3)

where *ETH*_*ij*_ is an indicator of the hourly close price of ETH at the *i*^*th*^ hour of the *j*^*th*^ day in Phase II. Similar definition could be written for the explanatory variable i.e., BTC. It is noteworthy to mention that the *j*^*th*^ profile is established at the end of the day once the 24^th^ candle is closed (there is not any profile in the middle of the day).

After receiving each profile at the end of a day as a pair of 24 dependent (ETH) and explanatory (BTC) variables (see Eq (3)), it is necessary to obtain a chart statistic based on the estimation of the parameters (see Eq (2)). To implement the CCPM for cryptocurrency data, two main challenges entailing random explanatory variables and existence of between and within profile autocorrelation occur.

There is a fundamental difference here with most of the existing researches in the area of profile monitoring such as Kang and Albin [26], Li and Wang [59], Mahmood, Riaz [60] and Abbasi, Yeganeh [61] as they have usually considered fixed (constant) explanatory variables in each profile (it means that the *j* index is omitted from Eq (1) in their approach). But this assumption is not valid in our problem since the BTC values are not constant in each day. There are few researches about linear profiles with random explanatory variables, one of them is Noorossana, Fatemi [46]. They extended some control charts, such as Hotelling’s T^2^, *F* test, EWMA and so forth, for this situation and evaluated their performance. Based on their results, the consideration of a random explanatory variable with the T^2^ control chart could be considered a proper choice for our problem. More details about implementation of the T^2^ control chart in simple linear profiles can be found in Kang and Albin [26] and Mahmoud and Woodall [62].

The second challenge is related to the existence of between and within profile autocorrelation. As mentioned by several authors [17,19], consideration of autocorrelation in financial data is a vital task and highly recommended. Two different autocorrelation effects are possible in the linear profiles, including between and within each profile. Some researchers extended proper control charts for each situation separately, two of which are Noorossana, Amiri [63] and Soleimani, Noorossana [64]. Our simulations revealed that the considered IC profile in Eq (3) had both autocorrelation effects simultaneously (the results are not given here but can be sent through on request). Hence, a novel T^2^ statistic by considering simultaneous autocorrelation effects is proposed here. The general idea is to combine the proposed approaches in Noorossana, Amiri [63] and Soleimani, Noorossana [64] which has been recently done by Ahmadi, Yeganeh [65].

To account for both auto-correlation effects in profile model through AutoRegressive time series of order one (AR(1)), Ahmadi, Yeganeh [65] proposed the following profile structure:

$$\begin{array}{l} ETH_{ij}=A_{0}+A_{l}BTC_{ij}+\varepsilon_{ij},\\ \varepsilon_{ij}=\rho \varepsilon_{(i-1)j}+a_{ij},\\ a_{ij}=\phi \varepsilon_{i(j-1)}+u_{ij},\\ i=1,2,\ldots ,24,\\ j=1,2,\ldots , \end{array}$$ (4)

where *ε*_*ij*_ and *a*_*ij*_ are the correlated error terms and *u*_*ij*_ are the independently andidentically normally distributed errors with mean zero and standard deviation *σ*. Furthermore, the explanatory variables *BTC*_*ij*_ are assumed to follow a normal distribution with mean *μ* and variance *σ*_*b*_^2^. In Eq (4), *ρ* and *ϕ* are the coefficients of AR(1) model in a way that *ρ* is used for the autocorrelation among the close hourly prices in each day (within-profile), while *ϕ* accounts for the autocorrelation among close prices of successive days (between-profile). Besides, to meet the weak stationarity of the AR models, we have | *ρ* |<1 and | *ϕ* |<1. From historical data, the values of *ϕ* and *ρ* in addition to the intercept, slope and standard deviation (*σ*) are estimated for each profile.

### 3.2. The proposed chart statistic in CCPM

To monitor the profile model in Eq (4) we decide to apply the Hotelling’s T^2^ control chart. To construct the chart statistic, some statistical results are needed. The proofs are neglected here for brevity, and readers are referred to Ahmadi, Yeganeh [65]. First, it could be easily shown that *E*(*ε*_*ij*_) = 0 and *V(ETH*_*ij*_*|BTC*_*ij*_ *= b*_*ij*_*) = V*(*ε*_*ij*_) = $\frac{\sigma^{2}}{\left(1‐\rho^{2})(1‐\phi^{2}\right)}$ where *b*_*ij*_ is the observed value of BTC price at the *i*^th^ hour of day *j*. Given *t* and *k* in a profile (it is obvious that *t ≠ k*), we obtain the conditional covariance between *ETH*_*tj*_ and *ETH*_*kj*_ given *b*_*ij*_ based on the covariance between *ε*_*tj*_ and *ε*_*kj*_ as *C*(*ETH*_*tj*_, *ETH*_*kj*_*|BTC*_*ij*_ *= b*_*ij*_) = *C*(*ε*_*tj*_, *ε*_*kj*_) = $\frac{\sigma^{2}\rho^{\left|k‐t\right|}}{\left(1‐\rho^{2})(1‐\phi^{2}\right)}$. On the other hand, according to Ahmadi, Yeganeh [65] and the discussion by Noorossana, Amiri [63] on the estimation of the profile parameters in the presence of random explanatory variables, it could be inferenced that $E({\hat{A}}_{0j}|BTC_{ij}=b_{ij})$ = *A*_0_ and $E({\hat{A}}_{1j}|BTC_{ij}=b_{ij})$ = *A*_*1*_ where ${\hat{A}}_{0j}$ and ${\hat{A}}_{1j}$ are be obtained by substituting *Y*_*ij*_ with *ETH*_*ij*_ and *X*_*ij*_ with *BTC*_*ij*_ in Eq (2). Eventually, we have:

$$\begin{array}{l} V({\hat{A}}_{0j}|BTC_{ij}=b_{ij})=\frac{\sigma^{2}}{\left(1-\phi^{2})(1-\rho^{2}\right)}(\sum_{i=1}^{n}q_{ij}^{2}+\sum_{t=1}^{n}\sum_{k=1}^{n}q_{ij}q_{kj}\rho^{\left|t-k\right|}),\\ V({\hat{A}}_{1j}|BTC_{ij}=b_{ij})=\frac{\sigma^{2}}{\left(1-\phi^{2})(1-\rho^{2}\right)}(\sum_{i=1}^{n}d_{ij}^{2}+\sum_{t=1}^{n}\sum_{k=1}^{n}d_{tj}d_{kj}\rho^{\left|-k\right|}),\\ C({\hat{A}}_{0j},{\hat{A}}_{1j}|BTC_{ij}=b_{ij})=\frac{\sigma^{2}}{\left(1-\phi^{2})(1-\rho^{2}\right)}\sum_{t=1}^{n}\sum_{k=1}^{n}q_{tj}d_{kj}\rho^{\left|t-k\right|}, \end{array}$$ (5)

where *q*_*ij*_ = $\frac{1}{n}‐\frac{{\bar{b}}_{j}(b_{ij}‐{\bar{b}}_{j})}{S_{j}}$, *d*_*ij*_ = $\frac{\left(b_{ij}-{\bar{b}}_{j}\right)}{S_{j}},$ *S*_*j*_ = $\sum_{i=1}^{n}{\left(b_{ij}-{\bar{b}}_{j}\right)}^{2}$ and ${\bar{b}}_{j}=\frac{1}{n}\sum_{i=1}^{n}b_{ij}$. Based on the above formula, the chart statistic at time *j* (*T*^*2*^_*j*_) is obtained as follows:

$$\begin{array}{l} T_{j}^{2}={\left[\left[{\hat{A}}_{0j}{\hat{A}}_{1j}]-[A_{0}A_{1}\right]\right]}^{T}\overset{-1}{\underset{j}{\sum }}[\left[{\hat{A}}_{0j}{\hat{A}}_{1j}]-[A_{0}A_{1}\right]],\\ \sum_{j}=(\begin{array}{cc} V({\hat{A}}_{0j}|BTC_{ij}=b_{ij}) & C({\hat{A}}_{0j},{\hat{A}}_{1j}|BTC_{ij}=b_{ij})\\ C({\hat{A}}_{0j},{\hat{A}}_{1j}|BTC_{ij}=b_{ij}) & V({\hat{A}}_{1j}|BTC_{ij}=b_{ij}) \end{array}). \end{array}$$ (6)

As mentioned by Noorossana, Fatemi [46], the variance-covariance matrix of the estimators is indicated by *Σ*_*j*_ that changes from each profile to another due to the randomness of explanatory variables.

### 3.3. The CCPM steps

The general steps of CCPM are defined in the subsections 3.3.1 to 3.3.3.

#### 3.3.1. Estimation step

This step, which is nearly equivalent to Phase I implementation in SPC, derives the IC profile parameters based on a N-days Previous Historical (NPH) data and also calculates the control limits. To this end, we have a dataset with NPH rows and 24 columns of hourly ETH and BTC close prices. The common approach for Phase I of profile monitoring is to obtain the outliers with T^***2***^ or F control charts [62] by considering a nominal false alarm rate and specifying the distribution of the chart statistic. Then the assignable causes for each outlier could be identified by conducting a proper diagnosis procedure. The outliers with sensible reasons are omitted from the historical data, while the others for which we are not able to find proper reasons are kept. Finally, the control limits are determined based on a specific type I error usually denoted by zero state average run length [26,66].

To follow the same manner, the proposed T^2^ statistic in Eq (6) is computed based on the *NPH* data (i.e., *j* = 1, 2, …, *NPH*). It is noteworthy to mention that IC parameters (*A*_*0*_, *A*_*1*_, *σ*, *ρ* and *ϕ*) are estimated based on the historical data in Phase I. Considering these values, *T*^*2*^_*j*_ is computed for each of the historical data. Finding an outlier in Phase I is a possible task in industry but as mentioned by some researches such as Garthoff, Golosnoy [23], Dumičić and Žmuk [24] and others, this approach is usually unachievable in financial market as we are not able to terminate the trading process in a market. On the other hand, the control limits entailing Lower Control Limit (LCL) and Upper Control Limit (UCL) of conventional T^2^ control charts in the previous researches such as Kang and Albin [26], Noorossana, Amiri [63] and Soleimani, Noorossana [64] were computed by chi-square distribution and it was shown that the Run Length (RL) had a geometric distribution. That said, it is wrong for our approach as the computed statistics are not independent here (due to between profile autocorrelation). So, the RL of T^2^ control charts does not follow the geometric distribution under our assumptions. For more details about RL distribution, one can refer to Knoth, Tercero-Gómez [66]. This problem was also discussed in Noorossana, Amiri [63] and they suggested a residual-based T^2^ control chart to tackle it.

To remedy these challenges, a nominal false alarm rate is assigned (denoted by *α*_*nom*_) and then, the 0.5*α*_*nom*_ and 1–0.5*α*_*nom*_ quantiles of *NPH T*^*2*^_*j*_ statistics are considered as the *LCL*_*T*_ and *UCL*_*T*_. The unstable profiles located beyond the limits (*T*^*2*^_*j*_ > *UCL*_*T*_ or *T*^*2*^_*j*_ < *LCL*_*T*_) are usually omitted in conventional Phase I methods and the IC parameters (*A*_*0*_, *A*_*1*_, *σ*, *ρ* and *ϕ*) are estimated based on the remaining profiles, by averaging from them. But we do not apply this step as we are not able to find true reason for the outliers in financial markets. Note that the conventional T^2^ control charts in Kang and Albin [26], Noorossana, Amiri [63] and Soleimani, Noorossana [64] do not have LCL but it is meaningful in CCPM.

#### 3.3.2. Monitoring step

This step is introduced based on the Phase II analysis in SPC. To this end, at the end of each day, the profile is first constructed and then, estimation of intercept (${\hat{A}}_{0j}$), slope (${\hat{A}}_{1j}$), and Σ_***j***_ are calculated. Then by Eq (6), the statistic is computed based on the IC parameters obtained from Phase I where index j is indicator of the j^***th***^ day from the beginning of the monitoring step. The signal is achieved in case T^***2***^_***j***_ > UCL_***T***_ or T^***2***^_***j***_ < LCL_***T***_. Here, there is another difference with the industrial process in which the process terminates with the first signal; note though, Hassan, Kumiega [22] recommended waiting for at least 5 OC profiles to interpret a signal. For this aim, we also define the Number of OC (NOC) consecutive profiles index as the number of OC consecutive profiles before a sell or buy signal. This means that locating at least (NOC + 1) consecutive T^***2***^_***j***_ statistics beyond the control limits is necessary in CCPM to terminate the monitoring step. Due to definition of NOC criteria, reaching a signal by LCL has an extremely low probability especially in the range market but it is expected to meet the condition in some special trends such as high decreasing [25].

#### 3.3.3. Decision making step

As there are (NOC + 1) OC profiles in this step, we can conclude that the process is unstable and tends to come back to its stable (IC) condition. In the financial market, the stable condition could be considered as a reference price. Suppose that at time S (S > NOC), the process of Phase II terminates by location of (NOC + 1) consecutive T^***2***^_***j***_ statistics beyond the control limits and we have S profiles. The average of ETH/BTC prices is calculated based on the S profiles by dividing the responses on explanatory variables in each hour of day (i.e., the average of 24 values is computed for each day and then average of S ETH/BTC prices is reached) we denote this as the Current Price (CP). Also, the Reference Price (RP) is obtained based on the profiles in the estimation step. The CP and RP are considered unstable and stable conditions, respectively. Our idea suggests that the future of the market moves from CP toward RP to reach the stable condition. It could be concluded that a relatively large decrease (increase) in the future price would be seen in case CP > RP (CP < RP); hence, the following equation represents our decision strategy:

$$\begin{array}{l} if\;CP>RP->Sell,\\ if\;CP<RP->Buy. \end{array}$$ (7)

**Fig 2:**
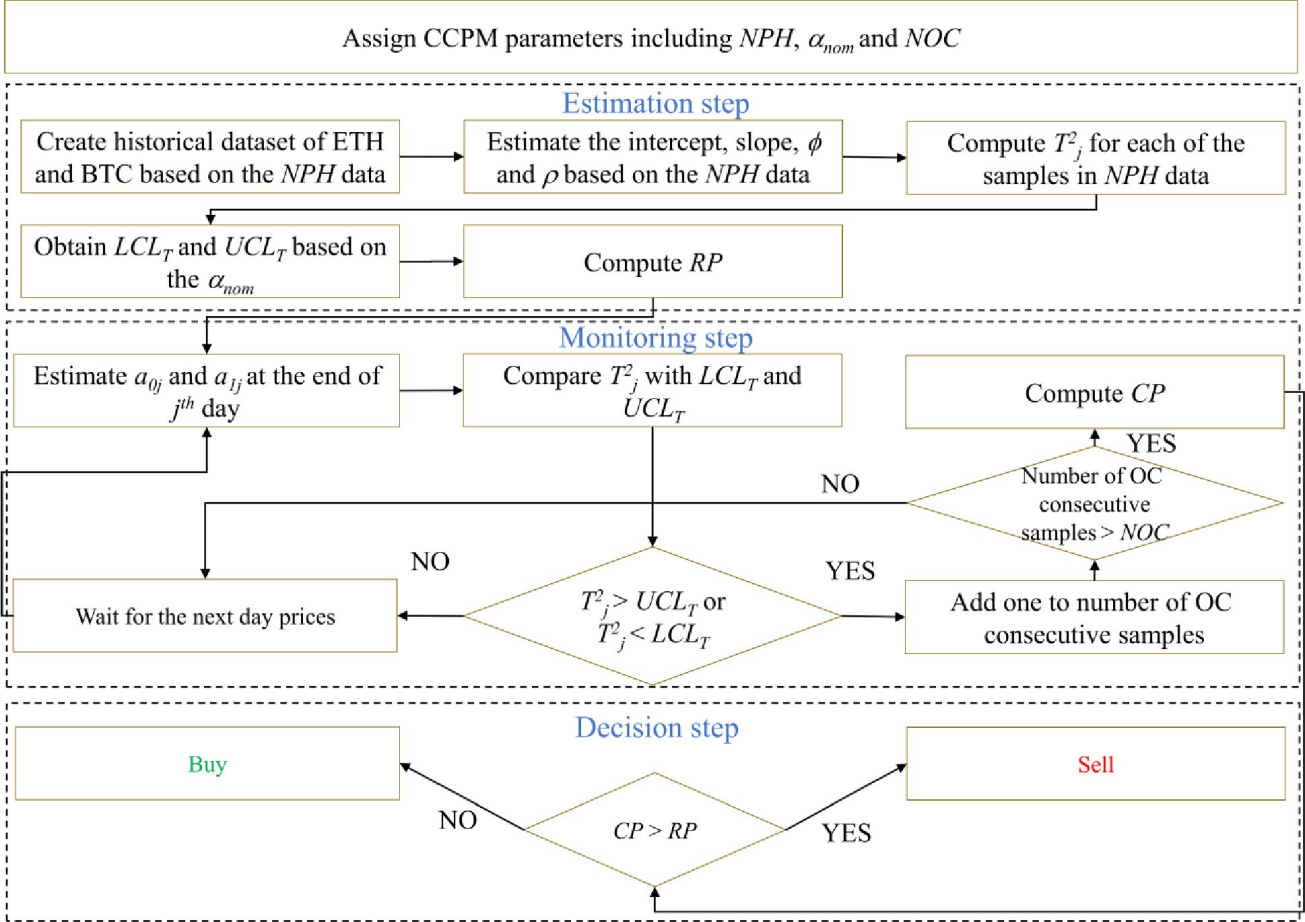
The framework of CCPM.

It is obvious that the decision here is equal to changing the assets from BTC to ETH (for buy) and ETH to BTC (for sell) as shown in Table 1. It is also noteworthy to mention that it is expected that we can reach our strategy sooner to a relatively large decrease (increase) in the future price in case of a sell (buy) decision. The latter is shown by the simulation in the next section. Note though, for a better understanding, Fig 2 illustrates the framework of the CCPM approach.

## 4. Simulation studies

This section provides a simulation analysis to illustrate the performance of the proposed technique. First, an example of a whole CCPM procedure is provided in Subsection 4.1. Then, the performance of CCPM under different parameter adjustments is investigated. Subsection 4.3 compares CCPM with other technical indicators. Finally, the performance of Hoteling T^2^ control chart are evaluated through simulation studies under three major Phase II criteria. For the simulation studies, the close hourly prices of BTC, ETH and ETH/BTC from 01/01/2019 to 07/06/2022 were extracted from https://www.cryptodatadownload.com (see Fig 1).

### 4.1. An example of CCPM procedure for a decision

To clearly illustrate the implementation procedure of the proposed CCPM, which is also depicted in Fig 2, let us consider the real data under the following setup:

- Current day: 26/02/2022, *NPH* = 40 days, *NOC* = 5 consecutive days, and *α*_*nom*_ = 0.1.

**Fig 3:**
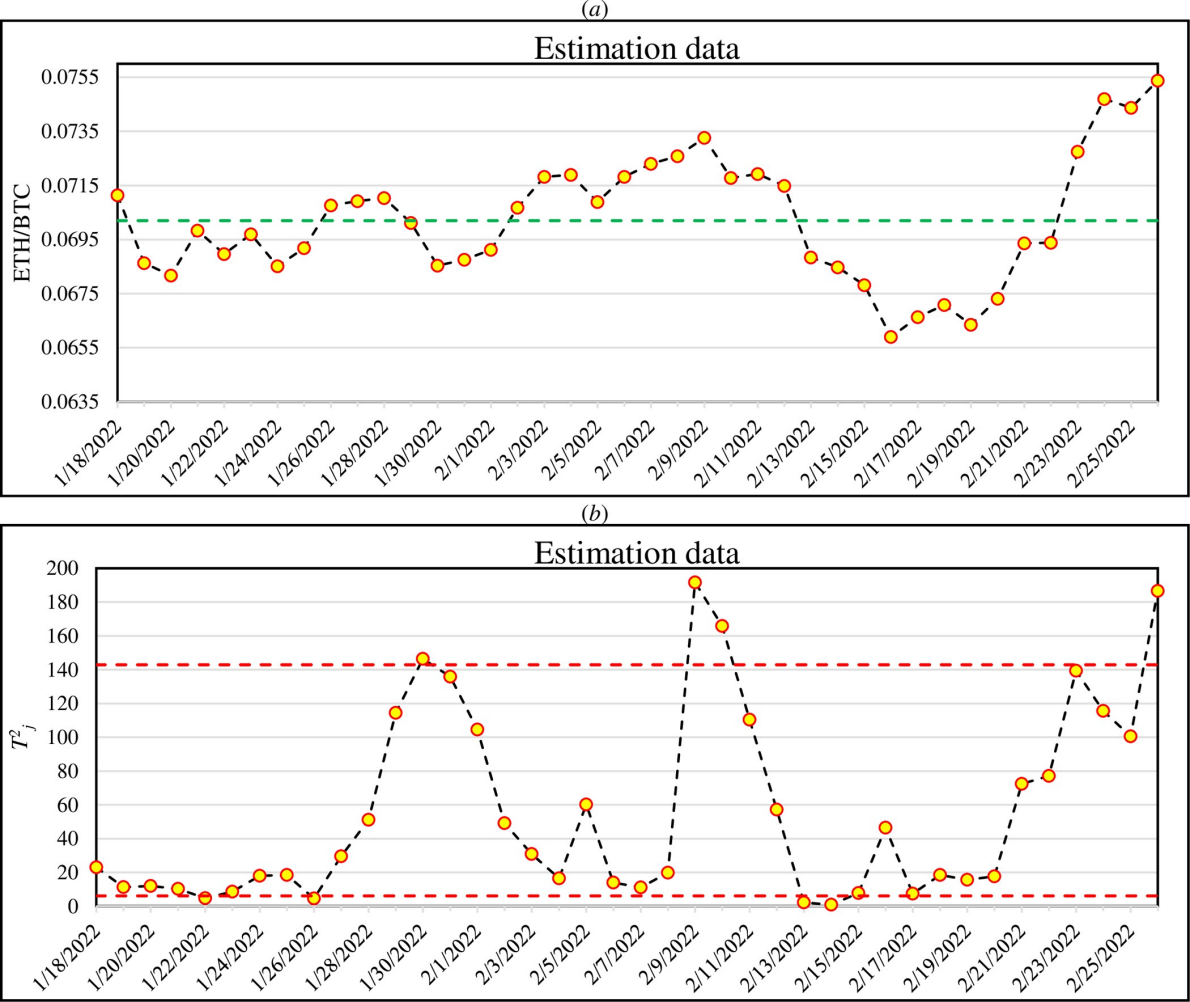
The average of hourly ETH/BTC prices in each day of estimation step (a) and obtained T^2^_j_ statistics (b). The current day is at the end of the chart (26/02/2022). The green line in panel (a) is the average of ETH/BTC prices in the estimation step which is called RP (0.0702). The red lines in panel (b) are the LCL_T_ and UCL_T_.

By setting *NPH* = 40 days, the estimation step includes the range 18/01/2022 to 26/02/2022 so that 40 profiles are involved in the estimation. Fig 3(A) depicts the average of ETH/BTC hourly prices in each day of estimation step in a way that each point is obtained by the averaging of 24-hourly prices. For each day in estimation step, a profile is constructed and the parameters are estimated. Following Eq (6), the *T*^*2*^_*j*_ statistics are obtained for each day and depicted in Fig 3(B). The green line in Fig 3(A) is the average of the ETH/BTC prices in the estimation step which is called *RP*. It was obtained as 0.0702. Also, the red lines in Fig 3(B) are *LCL*_*T*_ and *UCL*_*T*_ calculated based on the 0.5*α*_*nom*_ and 1–0.5*α*_*nom*_ quantiles of the computed *T*^*2*^_*j*_ statistics which are obtained as 6.15 and 142.91, respectively.

The normality of explanatory variable is checked by one-sample Kolmogorov-Smirnov test and then the estimated parameters based on the estimation data are obtained as *A*_*0*_ = -1150.6, *A*_*1*_ = 0.099, *ρ* = 0.033 and *ϕ* = 0.653. It is shown there is weak correlation within each profile or the prices in each hour of a day while it is tangible for the between profiles. Then, the *T*^*2*^_*j*_ statistics are computed for each day.

**Fig 4:**
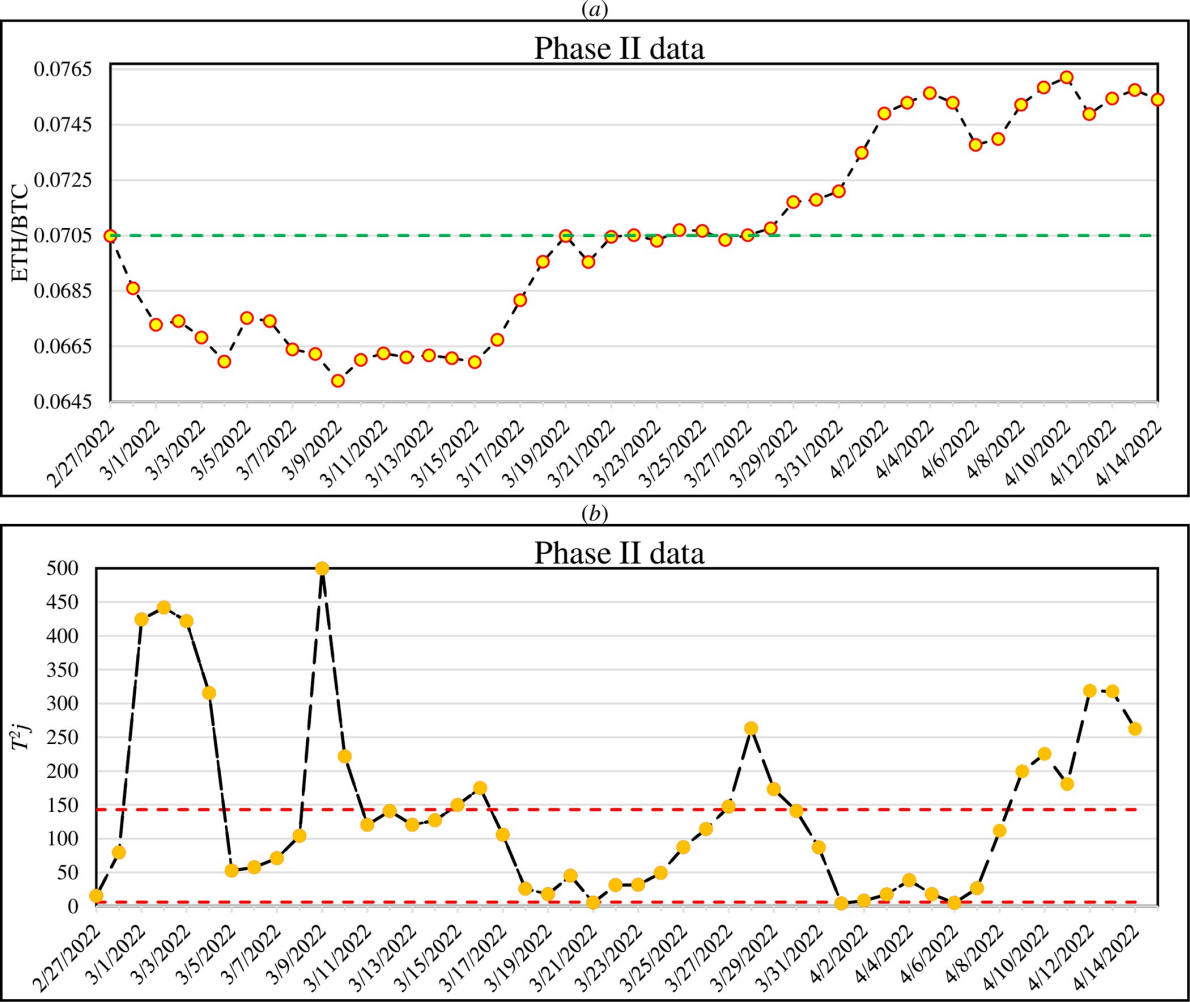
The average of hourly ETH/BTC prices in each day of Phase II data (a) and obtained T^2^_j_ statistics (b). Due to 6 (NOC + 1) consecutive signals, a decision about a buy or sell action is needed at 15/04/2022. The green line in panel (a) is the average of ETH/BTC prices in Phase II which is called CP (0.0705). The red lines in panel (b) are the LCL_T_ and UCL_T_.

Considering the IC parameters, the monitoring step (Phase II) is conducted by calculating *T*^*2*^_*j*_ statistics for the subsequent days after 26/02/2022. This means that *T*^*2*^_*j*_ calculated at the end of each day until reaching 6 (5+1) OC consecutive signals. This occurred 47 days later (*S* = 47), i.e., 15/04/2022 (note that the data of 29/02 to 31/02 is not available on the website). For a better illustration, Fig 4(A) shows the average of ETH/BTC hourly prices in *S* days of Phase II in a way that each point is calculated by the averaging of 24-hourly prices. For each day in Phase II, a profile is constructed and the parameters are estimated. Following Eq (6), the *T*^*2*^_*j*_ statistics are obtained for each day and depicted in Fig 4(B). The green line in Fig 4(A) is the average of the ETH/BTC prices in Phase II which is denoted as *CP*. It was computed by the *S* days data as 0.0705. Similar to Fig 3(B), the red lines in Fig 4(B) are *LCL*_*T*_ and *UCL*_*T*_.

As there are 6 consecutive OC signals, it is necessary to decide about a sell or buy action. Considering *CP* > *RF*, the CCPM method declares a sell action (see Eq (7)). In the stock market trading, it is common to define *Take Profit* and *Stop Loss* criteria to manage the technical indicators’ signals [67]. We follow similar idea to evaluate the performance of our proposed method. Similar to *Take Profit*, we define a Target Ratio (*TR*) as the specific relative decrease (increase) in ETH/BTC price based on the sell (buy) signal. It means that we receive the benefit of the trade when the ETH/BTC price has a decrease (increase) with a relative magnitude *TR* in a sell (buy) action. As a secondary criterion, it is favorable to reach the new price based on *TR* as soon as possible. For example, suppose that the CCPM indicates a sell signal; it is considered as a right signal if one can first reach to a *TR* relative decrease in the prices after the (*NOC* + 1) consecutive OC signals. Naturally, it is a false signal in case of a sooner occurrence of a relative increase with magnitude *TR*. To measure it, we count the number of required days to reach a relative decrease (in sell signal) or increase (buy signal) in ETH/BTC price with magnitude *TR*. In fact, it is utilized instead of *Stop Loss*.

**Fig 5:**
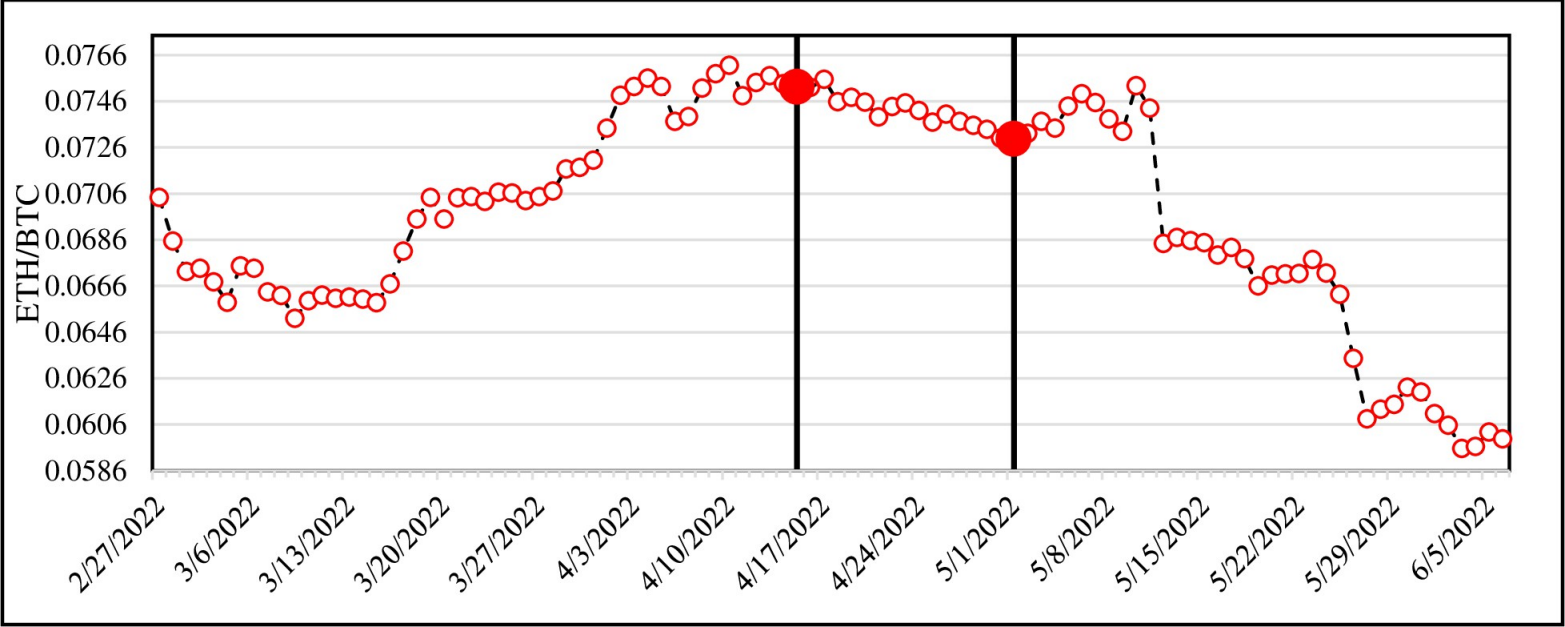
The average ETH/BTC hourly price after the end of Phase I to the end of dataset. The first vertical line is the time of a sell signal (15/04/2022) and the second indicates the time that the price relative decrement would be smaller than TR (01/05/2022). The subsequent days (after the second vertical line) are only plotted to show the general decreasing patterns.

After the end of Phase II and a sell action, indeed 15/04/2022, we check the needed time to reach a relative decrease under the assumption of *TR* = 0.03. It is found that 16 days later, i.e., on 01/05/2022, the ETH/BTC price was equal to 0.073; so, the relative decrease was lower than *TR* ($\frac{0.075–0.073}{0.075}$ = -0.03004). In other words, by selling our asset on 15/04/2022 (i.e., converting ETH to BTC) and buying at 01/05/2022 (i.e., converting BTC to ETH), we would acquire about 0.03 benefit within 16 days. This procedure is shown in Fig 5 in which the vertical lines are 15/04/2022 and 01/05/2022, respectively. It is obvious that the first 47 points in Fig 5 (i.e., before the first vertical line) are identical to those in Fig 4(A).

As it can be seen, CCPM with the estimated parameters had triggered a sell signal on the 47^th^ day of Phase II (15/04/2022) and the price relative decrement reached lower than 0.03 (*TR*) 16 days later (01/05/2022). For better investigation, we have plotted the prices until the end of our dataset and it could be said that there are no prices that would be relatively greater than *CP* with magnitude *TR* until 07/06/2022 (end of the dataset). So, the CCPM was able to provide a true signal. In this case, by increasing the absolute value of *TR*, we could get more benefits from 08/05/2022 to 05/06/2022; however, it may lead to improper decisions in other timestamps. We will discuss more about the effect of *TR* in the next subsections.

### 4.2. Sensitivity analysis about CCPM parameters

To reach the best performance with the CCPM, this subsection provides a sensitivity analysis of two main parameters including, *NPH* and *NOC*. It should be noted that we did not investigate *α*_*nom*_ as its effect was not very tangible. To this aim, a simulation study with 1000 iterations was defined. In each iteration, a timestamp from the dataset was randomly selected (in range 01/01/2019 to 07/06/2022 with consideration of *NPH* and *NOC*) and the three steps of CCPM were applied. Similar to subsection 4.1, the needed time to reach a relative *TR* decrease (increase) after a sell (buy) action was measured and considering this measurement, four criteria were defined. In some iterations, especially at the end of the dataset, a desired relative decrement (increment) in the price with the magnitude *TR* was not seen after a sell (buy) action. For example, the prices in the previous subsection have not reached an increase with magnitude *TR* after 15/04/2022 until the end of dataset. We showed them by the Ratio of Uncompleted Signals (*RUS*). If the CCPM approach triggered a buy action in the example of subsection 4.1, it would be considered an uncompleted signal. To compute *RUS*, the ratio of times (in 1000 iterations) that we could not reach the desired price based on our action is calculated. In addition to *RUS*, the number of days to reach the desired price after a signal was computed for each iteration (for example, it was 16 days in the previous subsection) and the average, standard deviation and median of them were shown by Average of Days to Desired Price (*ADDP*), Standard deviation of Days to Desired Price (*SDDP*) and Median of Days to Desired Price (*MDDP*), respectively. In other words, the procedure of subsection 4.1 is iterated 1000 times with random current time and specified values of *NPH* and *NOC*. Among them, the *RSU*×100% of the iterations’ results are omitted due to unavailability to reach the desired price and *ADDP*, *SDDP* and *MDDP* are obtained by the others. We considered nine different values of *TR* as 0.015, 0.03, 0.05, 0.07, 0.1, 0.12, 0.15, 0.3 and 0.4, each of which included an independent simulation study.

First, the effect of *NPH* was investigated by the above procedure. Ten values entailing 4, 8, 12, 16, 20, 24, 28, 35, 45 and 60 days were considered, and then, the simulation study with 1000 iterations was conducted for each of them. Table 2 reports the results of these situations. In each comparison, the best result was bolded. As the first obvious conclusion, it could be inferred that the larger TR values have increased all the criteria in most cases. It is a rationale finding as when a trader wants to reach more benefit (equivalently more *TR*), more time is needed (larger values of *ADDP*, *SDDP* and *MDDP*) and there is a higher risk of reaching a target price (larger value of *RUS*). As the secondary finding, it is not possible to select an exact superior value for *NPH* since the performances are different. But it might be said that moderate *NPH* values like 16 or 20 could be proper options for the low *TR* values whereas the greater *NPH* values the better performance in large *TR* values. For example, we can see superior performance in *TR* = 0.3 and 0.4 with *NPH* = 35 and 45.

**Table 2:** The sensitivity analysis of NPH through different values of TR.

*TR*	*RUS*	*ADDP*	*SDDP*	*MDDP*	*NPH*
0.015	0.21	22.99	37.63	6	4
0.03	0.14	27.97	33.51	13	4
0.05	0.15	25.05	27.71	16	4
0.07	0.19	29.77	31.06	19	4
0.1	0.26	41.19	36.54	25	4
0.12	0.26	42.84	33.56	32.5	4
0.15	0.43	**38.44**	**26.74**	32	4
0.3	0.62	83.03	37.69	79.5	4
0.4	0.68	127.25	75.52	127	4
0.015	0.12	20.40	32.70	5.5	8
0.03	0.22	23.60	33.52	7.5	8
0.05	0.22	23.08	**24.78**	12.5	8
0.07	0.23	29.47	27.36	19	8
0.1	0.22	37.45	33.13	28.5	8
0.12	0.25	**37.52**	33.48	27	8
0.15	0.45	41.51	32.83	**31**	8
0.3	**0.55**	68.40	33.72	61	8
0.4	0.67	117.18	75.03	100	8
0.015	0.10	22.61	36.93	7	12
0.03	0.09	32.80	46.22	9	12
0.05	0.22	26.94	30.65	13	12
0.07	0.2	25.58	**22.71**	20	12
0.1	0.32	**30.01**	25.92	23	12
0.12	0.33	42.03	38.13	30	12
0.15	0.32	47.10	32.66	37.5	12
0.3	0.64	67.47	32.29	62.5	12
0.4	0.74	113.23	67.29	93.5	12
0.015	0.08	17.84	**28.34**	7	16
0.03	0.14	**18.57**	**28.79**	**6**	16
0.05	0.2	**20.58**	26.04	**10.5**	16
0.07	0.25	29.91	27.44	20	16
0.1	**0.21**	31.81	26.63	**23**	16
0.12	0.22	40.10	32.27	30	16
0.15	0.3	41.30	29.04	37	16
0.3	0.67	74.79	40.83	64	16
0.4	0.66	81.00	58.05	53.5	16
0.015	0.11	**15.76**	31.03	**5**	20
0.03	0.11	24.40	37.78	7	20
0.05	0.16	30.38	32.10	17.5	20
0.07	0.13	25.32	26.88	14	20
0.1	0.24	31.12	**24.24**	26.5	20
0.12	0.24	41.28	31.99	35	20
0.15	0.3	40.20	28.88	33	20
0.3	0.62	63.87	39.93	48	20
0.4	0.66	119.71	81.54	105.5	20
0.015	**0.07**	21.72	38.27	6	24
0.03	0.18	25.11	39.78	6.5	24
0.05	0.19	24.52	31.21	12	24
0.07	**0.12**	32.42	35.15	19	24
0.1	0.23	33.84	31.06	25	24
0.12	0.26	37.70	**31.01**	30	24
0.15	0.29	39.30	30.50	33	24
0.3	0.63	65.81	33.77	57	24
0.4	0.67	82.00	62.80	56	24
0.015	0.14	18.41	34.15	5	28
0.03	0.14	31.51	48.13	9	28
0.05	**0.11**	25.51	30.68	14	28
0.07	0.14	**24.81**	28.51	**13**	28
0.1	0.24	40.62	34.90	28.5	28
0.12	0.25	40.93	35.65	35	28
0.15	0.29	54.15	43.59	37	28
0.3	0.62	60.87	36.47	49	28
0.4	0.75	86.28	69.97	50	28
0.015	0.11	22.37	36.41	8	35
0.03	0.12	27.99	47.85	7	35
0.05	0.17	25.46	33.92	12	35
0.07	0.15	34.04	30.23	25	35
0.1	0.23	40.40	35.59	25	35
0.12	0.26	38.95	33.60	30	35
0.15	0.24	52.33	43.05	37	35
0.3	0.62	**49.95**	**31.46**	**43**	35
0.4	**0.65**	79.71	53.28	70	35
0.015	0.16	20.13	31.78	6.5	45
0.03	**0.08**	23.96	35.29	7	45
0.05	0.19	33.02	36.50	14	45
0.07	0.25	32.57	28.17	25	45
0.1	0.24	39.14	38.07	26	45
0.12	**0.18**	39.35	37.65	**26.5**	45
0.15	0.28	51.49	39.52	37	45
0.3	0.62	66.50	44.78	54.5	45
0.4	0.68	**58.06**	41.86	**46**	45
0.015	0.15	18.93	29.91	8	60
0.03	0.11	25.80	31.66	11	60
0.05	0.17	28.40	30.44	17	60
0.07	0.21	33.82	34.40	24	60
0.1	0.27	39.18	32.02	26	60
0.12	0.25	45.24	39.57	30	60
0.15	**0.18**	47.94	44.10	32	60
0.3	0.67	64.12	40.91	63	60
0.4	0.68	58.50	**37.64**	63.5	60

In the second experiment, the effect of *NOC* was investigated through nine different values of *TR*. Considering the same criteria as Tables 2 and 3 provides the accuracy results of the simulations for the sensitivity analysis about the *NOC* parameter with the values 4, 8, 12, 16, 20 and 24. An interesting finding is that the *ADDP*, *SDDP* and *MDDP* do not have a direct relation with *NOC*. In other words, it is expected that the needed days to reach the desired price is increased with larger *NOC* (as we should wait for more signals) but it can be seen from Table 3 that the results sometimes have a reverse relation. For example, in *TR* = 0.015, *ADDP* was obtained as 21.36 and 16.32 in *NOC* = 4 and 8, respectively. It could be justified from more OC profile and process instability point of view. As there are more OC profiles with larger *NOC*, the process has more instability so it might have more desire to come back to its stable (IC) condition. So, comparing small and moderate *NOC*, lower *ADDP*, *SDDP* and *MDDP* values were obtained by larger *NOC*. On the other hand, more time is needed to reach a signal in large *NOC* such as 20 and 24; hence the *RUS* was increased and it affected *ADDP*, *SDDP* and *MDDP*.

**Table 3:** Sensitivity analysis of NOC through different values of TR.

*TR*	*RUS*	*ADDP*	*SDDP*	*MDDP*	*NOC*
0.015	0.09	21.36	32.56	9	4
0.03	0.1	26.06	34.81	12	4
0.05	0.16	30.27	40.98	12.5	4
0.07	0.18	34.01	31.02	24.5	4
0.1	0.29	44.68	41.29	25	4
0.12	0.3	49.17	38.64	37.5	4
0.15	0.44	50.79	35.02	43	4
0.3	0.64	76.31	49.02	54	4
0.4	**0.62**	110.66	65.09	105.5	4
0.015	0.07	16.32	**25.00**	5	8
0.03	0.19	26.48	35.23	15	8
0.05	0.18	29.09	37.95	12.5	8
0.07	0.19	29.98	27.72	**20**	8
0.1	**0.19**	40.11	33.28	26	8
0.12	0.22	41.06	34.37	32.5	8
0.15	0.38	52.02	38.38	40	8
0.3	**0.59**	72.59	35.21	69	8
0.4	0.63	106.16	71.61	75	8
0.015	0.09	16.13	32.44	4	12
0.03	0.12	25.02	44.27	7	12
0.05	0.18	26.71	34.18	12	12
0.07	0.22	22.59	**21.82**	16.5	12
0.1	0.25	30.40	**22.46**	26	12
0.12	0.31	34.84	30.77	27	12
0.15	**0.23**	39.40	28.57	**31**	12
0.3	0.6	**64.30**	**30.41**	52	12
0.4	0.73	101.89	77.85	71	12
0.015	**0.05**	**11.41**	27.32	4	16
0.03	0.12	20.36	32.30	8	16
0.05	0.15	19.59	31.75	**10**	16
0.07	0.24	30.79	29.03	18.5	16
0.1	0.26	**28.22**	22.89	20	16
0.12	0.25	**32.64**	31.37	**21**	16
0.15	0.37	**34.63**	**24.33**	34	16
0.3	0.66	69.15	40.52	51.5	16
0.4	0.66	71.26	46.93	**54**	16
0.015	0.07	18.59	32.38	**3**	20
0.03	0.14	**17.90**	32.15	**4.5**	20
0.05	0.15	**19.31**	**24.81**	**10**	20
0.07	0.14	**21.07**	23.44	**13.5**	20
0.1	0.27	29.88	29.71	22	20
0.12	**0.2**	35.68	**26.91**	31	20
0.15	0.29	40.68	32.11	34	20
0.3	0.65	66.69	49.19	**45**	20
0.4	0.7	97.70	61.54	80.5	20
0.015	0.09	22.18	32.19	7	24
0.03	**0.08**	18.75	**31.98**	7	24
0.05	**0.14**	22.73	25.28	14	24
0.07	**0.11**	27.66	32.35	14	24
0.1	0.23	30.84	31.97	22	24
0.12	0.24	32.86	33.89	24	24
0.15	0.36	41.25	31.60	32	24
0.3	0.7	68.83	38.51	67.5	24
0.4	0.74	**70.92**	**44.30**	71	24

### 4.3. Comparing with the conventional technical indicators

As the idea of CCPM could be considered as a technical indicator, its performance was also compared with two conventional indicators i.e., RSI and MACD discussed in subsection 2.3. We implemented the same approach as subsections 4.1 and 4.2. After selecting a random date, the RSI and MACD were computed for the current time considering *NPH* data (‘*rsindex*’ and ‘*macd*’ MATLAB functions were used). For RSI, the value larger (lower) than 50 was considered a sell (buy) signal. Also, a sell (buy) signal was triggered when MACD line was located lower (above) than the signal line. Based on the previous results, we adjusted *NPH* and *NOC* as 20 and 10 in this subsection, where other adjustments and criteria were similar to the previous subsections.

Table 4 reports the performance comparison results of MACD, RSI and CCPM approaches under different values of *TR*. The superiority of CCPM over two other competitors is obvious in a wide range of *TR* values; for example, in *TR* = 0.05, *ADDP* was obtained as 23.96, 26.62 and 25 in CCPM, RSI and MACD, respectively. On the other hand, CCPM was able to reach the minimum *ADDP* (66.16 and 86.38) in the last two large *TR* values. So, it can be generally said that CCPM could provide superior results against two other competitors.

**Table 4:** The performance of MACD, RSI and CCPM (NPH and NOC were set at 20 and 10 respectively) approaches through different values of TR.

*TR*	*RUS*	*ADDP*	*SDDP*	*MDDP*	*Method*
0.015	**0.08**	**14.41**	27.82	**4**	MACD
0.03	**0.05**	**23.89**	37.65	**9**	MACD
0.05	0.19	25.00	32.22	12	MACD
0.07	0.21	36.95	47.65	**15**	MACD
0.1	**0.19**	**30.40**	39.84	**18**	MACD
0.12	**0.19**	**31.86**	32.22	**22**	MACD
0.15	0.35	48.25	46.79	**28**	MACD
0.3	0.52	76.50	53.04	62	MACD
0.4	**0.57**	98.16	77.26	99	MACD
0.015	0.11	18.02	**26.60**	5	RSI
0.03	0.18	27.98	34.46	13.5	RSI
0.05	0.19	26.62	34.89	**11**	RSI
0.07	**0.15**	40.89	39.92	24	RSI
0.1	0.27	38.78	38.90	23	RSI
0.12	0.32	48.60	44.85	**29**	RSI
0.15	0.45	**42.26**	29.42	**36**	RSI
0.3	0.54	81.11	44.94	72	RSI
0.4	0.67	107.09	71.59	91	RSI
0.015	0.08	17.28	28.63	8	CCPM
0.03	0.13	24.20	36.37	**9**	CCPM
0.05	**0.11**	**23.96**	**27.91**	**11**	CCPM
0.07	0.19	**26.84**	**25.03**	20	CCPM
0.1	0.32	38.06	**30.67**	27	CCPM
0.12	0.23	38.32	**29.43**	30	CCPM
0.15	**0.29**	47.65	**37.81**	37	CCPM
0.3	**0.43**	**66.16**	**40.10**	**51**	CCPM
0.4	0.68	**86.38**	**67.02**	**73**	CCPM

**Fig 6:**
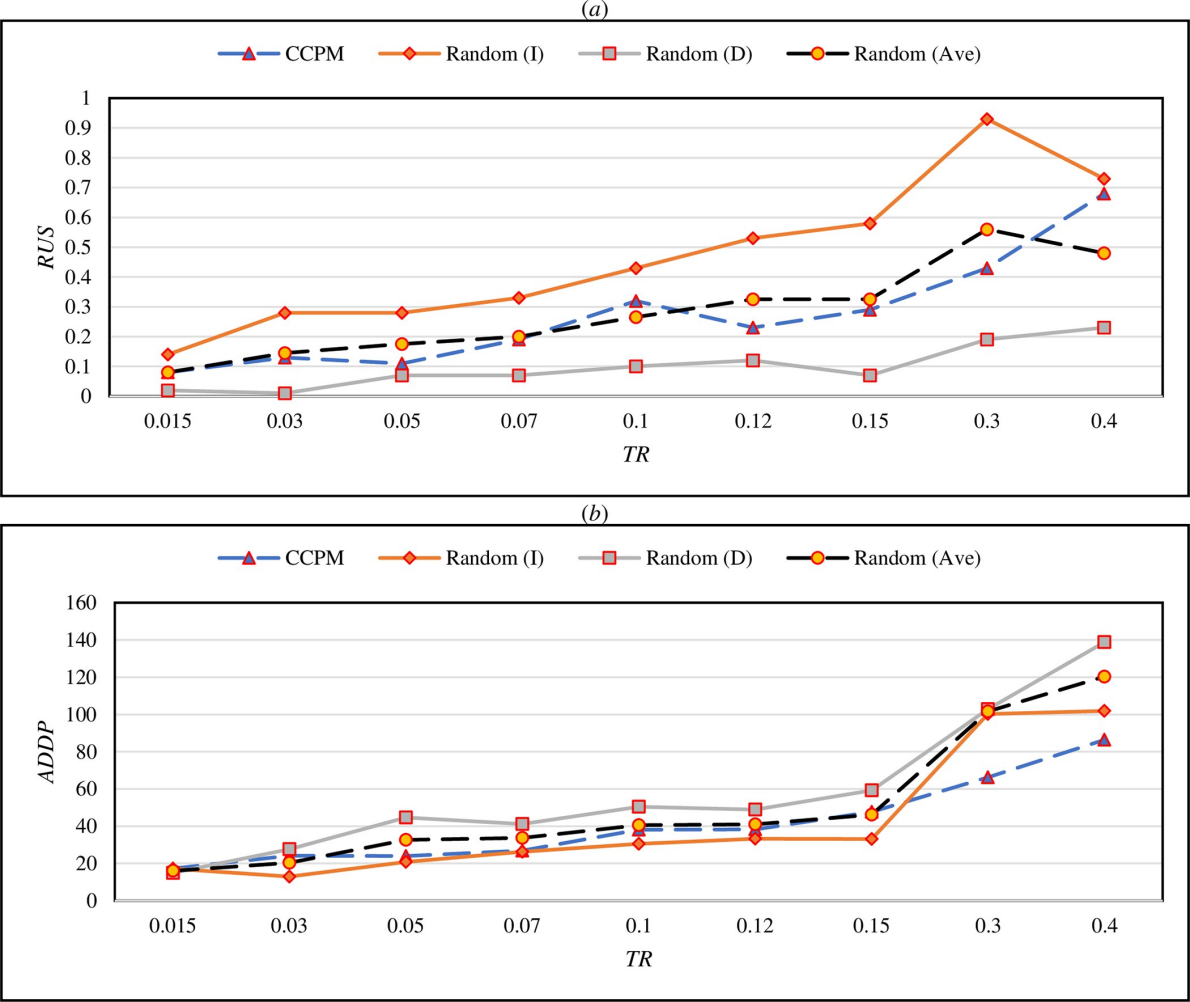
Comparing CCPM with random strategy in term of RUS (a) and ADDP (b) criteria.

One may be interested in the performance of a random strategy in which a trader randomly sells (buys) some crypto and waits for a specific decrease (increase) in its price. As another performance comparison, CCPM was evaluated with a random sell or buy strategy. In this approach, in a specific random timestamp, we randomly chose a buy (sell) action and then waited to reach an increase (decrease) with magnitude *TR*. They were denoted with Random (I) and Random (D). The results of *RUS* and *ADDP* are illustrated in Fig 6(A) and 6(B), respectively, through different values of *TR*. But it is not a fair comparison as we do not know the market’s direction and it is also not rational to use only buy or sell in all the trades. So, we got the average of the performances of Random (I) and Random (D) denoted by Random (Ave). As can be seen, the line of CCPM was located between Random (I) and Random (D) in both Fig 6(A) and 6(B) in such a way that Random (D) (Random (I)) had a better (worst) performance in term of *RUS* while CCPM was better (worse) than it in terms of *ADDP*, respectively. As a fairer comparison, CCPM reached lower *RUS* and *ADDP* than Random (Ave) in most *TR* values.

It could be generally inferenced from the above results that CCPM has a valuable performance as a technical indicator and could prompt a cascade of benefits for the professional traders in the cryptocurrency market. By this scheme, a trader can increase the total value of a pair asset in a shorter period than conventional technical indicators. Due to its remarkable performance, applying CCPM in other financial markets such as FOReign EXchange (FOREX), gold, petroleum and so forth is recommended as the future research suggestion. Also, investigation of other profile models such as polynomial, mixture, logistic, Poisson and so forth may lead to valuable findings in this field of study.

### 4.4. Simulation study for Phase II analysis

Three major criteria including Average Run Length (ARL), Standard Deviation Run Length (SDRL) and Median Run Length (ARL) are used to evaluate control charts in Phase II. They measure the required time to reach a signal on average. Naturally, lower criteria indicate a superior control chart. It is common to compute these performance criteria through Monte Carlo simulations when the exact distribution of RL is not known [36,68]. Considering the IC parameters, 10000 Monte Carlo simulations were performed to reach the three performance criteria. In these simulations, the shifts are added to the IC model in term of standard deviation units and then, the Hoteling T^2^ control chart is utilized to reach an OC signal. It is noteworthy to mention that both within and between auto-correlation coefficients are also considered in the generation of simulated profiles. In Table 5, the values of ARL, SDRL and MRL for shifts in the intercept, slope and standard deviation parameters are reported.

**Table 5:** The performance of Hoteling T^2^ control chart in the existence of within and between profile auto-correlation in term of ARL, SDRL and MRL criteria when there are artificial shifts in intercept, slope and standard deviation parameters.

Shift Size	Intercept			Shift Size	Slope			Shift Size	Standard deviation		
	ARL	SDRL	MRL		ARL	SDRL	MRL		ARL	SDRL	MRL
0.2	168.4	160.1	150.0	0.025	178.4	170.3	168.5	1.1	80.6	86.4	78.5
0.4	117.3	121.6	110.5	0.05	145.7	142.6	125.5	1.2	40.7	43.7	40.0
0.6	79.3	75.5	70.5	0.075	97.4	89.5	94.5	1.3	25.6	25.7	25.5
0.8	50.5	47.5	42.5	0.1	64.2	60.7	60.5	1.4	16.5	16.0	15.5
1	31.9	30.0	26.5	0.125	57.0	52.2	53.0	1.5	11.8	11.9	11.5
1.2	18.0	16.5	15.5	0.15	45.2	44.6	41.5	1.6	8.0	8.0	7.5

As expected, the quicker detection ability or lower performance criteria was obtained by larger shift magnitudes. Comparing different parameters, standard deviation was identified sooner than intercept and slope. The reason may be due to fewer impact of auto-correlation in the standard deviation parameter as similar results were also reported by Noorossana, Amiri [63], Soleimani, Noorossana [64] and Ahmadi, Yeganeh [65].

## 5. Concluding remarks

This paper proposes a novel application of SPC control charts in the financial market surveillance. Using the idea of profile monitoring, a novel control chart is extended to simultaneously monitor a pair of well-known cryptocurrencies i.e., BTC and ETH. In this approach, buy and sell signals which are equivalent to changing the assets from BTC to ETH (for buy) and ETH to BTC (for sell) are received from the proposed method called Control Chart based Profile Monitoring (CCPM). Then, the signals are interpreted based on the existing patterns in the historical data to reach a buy or sell decision. As there is a strong auto-correlation effect in the financial market data, a novel T^2^ control chart is utilized in this approach to consider the effect of between and within autocorrelation in the profiles. Since this idea provides a trading strategy based on the SPC charts, its performance is evaluated through some financial approaches and compared with conventional technical indicators. The results reveal that CCPM has a valuable performance as a technical indicator and could provide some benefits for the professional traders in the cryptocurrency market. For the future studies, application of CCPM on other financial markets such as FOREX, gold, petroleum and so forth is recommended. Also, the identification of the shifted profile parameters with profile diagnosis techniques is a worthwhile future research idea to pursue as it may lead to some new findings in signal interpretation and could be considered.

## Supporting information

S1 File (RAR)
